# Acquired double pylorus secondary to a completely epithelialized gastroduodenal fistula

**DOI:** 10.1055/a-2427-9263

**Published:** 2024-10-16

**Authors:** Cong Yuan, Fan Liu, Chun-Hui Xi, Ke Pu, Guodong Yang, Xian-Fei Wang, Xue-Mei Lin

**Affiliations:** 1 117913 Department of Gastroenterology, Affiliated Hospital of North Sichuan Medical College, Nanchong, China; 2 117913 Digestive Endoscopy Center, Affiliated Hospital of North Sichuan Medical College, Nanchong, China; 3 74655 Department of Pathology, Institute of Basic Medicine and Forensic Medicine, North Sichuan Medical College, Nanchong, China; 4 117913 Department of Pathology, Affiliated Hospital of North Sichuan Medical College, Nanchong, China

A 74-year-old man underwent a gastroscopic reevaluation due to a prior history of gastric antral ulcers two years ago ( Fig. 1 **a** ). The carbon-13 urea breath test was negative. The patient had taken a non-steroidal anti-inflammatory drug (diclofenac sodium) and proton pump inhibitors (omeprazole) periodically for back pain and ceased the NSAID six months ago. Esophagogastroduodenoscopy showed two pyloric channels that were communicating the gastric antrum with the duodenal bulb ( Fig. 1 **b** , Video 1 ). The endoscope could be passed into the duodenum through either channel. The channel located on the side of the lesser curvature, although fully epithelialized, had failed to contract, suggesting an accessory pylorus ( Fig. 2 **a, b** ). This patient was clinically asymptomatic but at risk for ulcer recurrence, and endoscopic follow-up was continued.

**Fig. 1:**
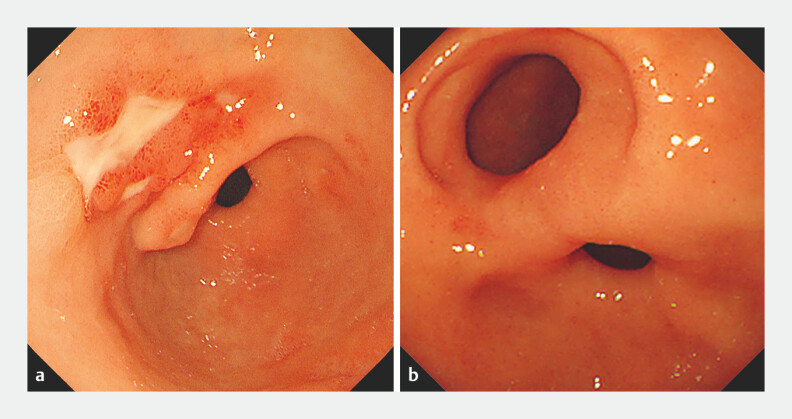
Endoscopic images of gastric antral ulcer progression. **a** The ulcers on the lesser curvature of the gastric antrum two years prior. **b** These ulcers developed into an antroduodenal fistula with a larger size than the anatomic pylorus.

Esophagogastroduodenoscopy showed that the ulcers on the lesser curvature of the antrum evolved into a completely epithelialized antroduodenal fistula with the absence of contraction. Acquired double pylorus was diagnosed.Video 1

**Fig. 2:**
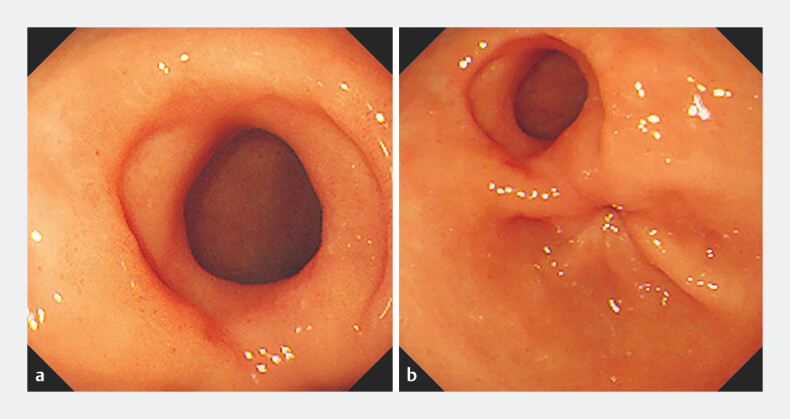
Endoscopic view of antroduodenal fistula and anatomic pylorus. **a** The antroduodenal fistula was completely epithelialized. **b** Anatomic pylorus showed normal contractile function.

Double pylorus is generally an incidental finding with a reported endoscopic incidence of between 0.001% and 0.08% 1 . It is rarely congenital 1 , or more commonly acquired as a complication of a prepyloric, duodenal ulcer or gastric carcinoma that perforates the gastric and duodenal walls over time and creates an antroduodenal fistula 2 3 . Most fistulas are located on the lesser curvature of the gastric antrum, followed by the posterior wall, greater curvature, and anterior wall 4 . Some patients have a predisposing factor of ulcerogenic drug use or *Helicobacter pylori* infection. Multiple systemic diseases, such as diabetes, liver cirrhosis, and chronic obstructive pulmonary disease, may be associated with acquired double pylorus. The accessory orifice with an absence of normal contraction can cause reflux of duodenal contents 4 . The natural history of the fistula is variable, with approximately 60% remaining patent and a few progressing to three pyloric ostia, spontaneous closure, or fusion into a large single pylorus 3 4 5 . Double pylorus is usually treated conservatively and only in rare cases is surgical intervention required.

Endoscopy_UCTN_Code_CCL_1AB_2AD_3AC

## Notes

Endoscopy E-Videos https://eref.thieme.de/e-videos
*E-Videos* is an open access online section of the journal *Endoscopy* , reporting on interesting cases and new techniques in gastroenterological endoscopy. All papers include a high-quality video and are published with a Creative Commons CC-BY license. Endoscopy E-Videos qualify for HINARI discounts and waivers and eligibility is automatically checked during the submission process. We grant 100% waivers to articles whose corresponding authors are based in Group A countries and 50% waivers to those who are based in Group B countries as classified by Research4Life (see: https://www.research4life.org/access/eligibility/ ). This section has its own submission website at https://mc.manuscriptcentral.com/e-videos .
